# Risk of gestational hypertension-preeclampsia in women with preceding endometriosis: A nationwide population-based study

**DOI:** 10.1371/journal.pone.0181261

**Published:** 2017-07-17

**Authors:** Mei-Lien Pan, Li-Ru Chen, Hsiao-Mei Tsao, Kuo-Hu Chen

**Affiliations:** 1 Institute of Information Science, Academia Sinica, Taipei, Taiwan; 2 Department of Physical Medicine and Rehabilitation, Mackay Memorial Hospital, Taipei, Taiwan; 3 Department of Mechanical Engineering, National Chiao-Tung University, Hsinchu, Taiwan; 4 Department of Obstetrics and Gynecology, Taipei Tzu-Chi Hospital, Buddhist Tzu-Chi Medical Foundation, Taipei, Taiwan; 5 School of Medicine, Tzu-Chi University, Hualien, Taiwan; John Hopkins University School of Medicine, UNITED STATES

## Abstract

**Objective:**

To investigate the association between preceding endometriosis and gestational hypertension-preeclampsia (GH-PE).

**Methods:**

In this nationwide population-based longitudinal study, data from 1998–2012 Taiwan National Health Insurance Research Database were used. We used ICD9-CM codes 617.X and 642.X respectively for the diagnoses of endometriosis and GH-PE, which were further confirmed by examining medical records of surgeries, blood pressure and urine protein to ensure the accuracy of the diagnoses. The study excluded women diagnosed with endometriosis at < 15 or > 45 years of age, chronic hypertension, and GH-PE prior to endometriosis. Each pregnant woman with a prior diagnosis of endometriosis was matched to 4 pregnant women without endometriosis by age. Logistic regression analysis was used to calculate odds ratios (ORs) for the risk of GH-PE with adjustment for age, occupation, urbanization, economic status and comorbidities.

**Results:**

Among 6,300 women with a prior endometriosis diagnosis who were retrieved from a population of 1,000,000 residents, 2,578 (40.92%) had subsequent pregnancies that were eligible for further analysis and were compared with 10,312 pregnant women without previous endometriosis. GH-PE occurred more in women with prior endometriosis as compared to those without endometriosis (3.88% vs. 1.63%, *p*<0.0001). Further analysis revealed prior endometriosis was associated with GH-PE (adjusted OR = 2.27; 95% CI:1.76–2.93). For danazol-treated and non-danazol-treated subgroups, the incidences of GH-PE were 3.13% (15/480) and 4.05% (85/2,098), respectively. Although the risk for subsequent GH-PE was lower (adjusted OR = 1.49; 95% CI:0.86–2.56) after receiving danazol treatment than average (adjusted OR = 2.27; 95% CI:1.76–2.93) for women with preceding endometriosis, the reduction of risk was not statistically remarkable for danazol-treated (adjusted OR = 1.49) vs. non-danazol-treated (adjusted OR = 2.48) subgroups (p heterogeneity = 0.12).

**Conclusions:**

Preceding endometriosis is an independent and significant risk factor for the occurrence of GH-PE.

## Introduction

Endometriosis, a common cause of pelvic pain and subfertility, is characterized by endometrial-like tissue outside the endometrium, primarily on the uterine myometrium, ovaries, rectovaginal septum and pelvic peritoneum [1]. The prevalence of endometriosis is estimated to be approximately 6%-10% of reproductive-aged women [1, 2] Although the etiology and pathogenesis of endometriosis remain uncertain, the ectopic endometrial cells and tissues, which are dependent on estrogen for growth, can implant on peritoneal surfaces and elicit a chronic inflammatory response and subsequent adhesions, fibrosis, scarring, neuronal infiltration, and anatomical distortion [1–3]. Invasion of the peritoneal epithelium is postulated to be mediated by an immune response, with subsequent activation of macrophages, increased production of cytokines, growth factors, and angiogenic factors [2]. In contrast, natural-killer-cell (NK cell) activity in peritoneal fluid in women with endometriosis is compromised, which can lead to decreased surveillance of ectopic tissue [2].

Hypertension is the most common medical complication of pregnancy. Approximately 70% of women diagnosed with a hypertensive disorder during pregnancy will have gestational hypertension/preeclampsia (GH-PE), with an overall prevalence of 6%-8% [4]. GH or pregnancy-induced hypertension is defined as a systolic blood pressure (BP) ≥ 140 mmHg or a diastolic BP ≥ 90 mmHg after 20 weeks of gestation without the presence of proteinuria [4, 5]. Some women with GH will subsequently progress to PE, which affects 2%–5% of pregnancies and is traditionally diagnosed by the presentation of high BP (≥ 140/90 mmHg), accompanied by proteinuria (≥ 300 mg /24 h or ≥ 1+ on a dipstick) or maternal organ dysfunction [4–8]. PE is one of the main causes of maternal and perinatal morbidity and mortality, especially in low- and middle-income countries [5, 6, 8], and predisposes mothers and fetuses to cardiovascular disease later in life [6, 7, 9–11]. Common risk factors for GH-PE include older age, first pregnancy, pre-pregnancy obesity (elevated body mass index [BMI]), multiple-fetus pregnancy, polycystic ovarian syndrome, chronic kidney disease, overt diabetes, and autoimmune disease [4–7, 12–18]. However, in clinical practice, these risk factors only predict 30% of women who will develop PE [6–7]. The two-stage model of abnormal trophoblastic invasion during placental implantation, placental ischemia, and subsequent endothelial injury has been established as the possible etiology and pathophysiology underlying GH-PE [4, 5, 7, 12, 19, 20]. Some biophysical and biochemical methods including maternal uterine artery Doppler [7, 9, 13, 19], fetal aorta intima-media thickness (aIMT) [9], and biochemical markers [12–14, 18] have been developed for detecting PE among low-risk populations. Nevertheless, much remains unknown regarding the mechanisms and markers of GH-PE.

Even though the relationship between endometriosis and infertility is well established, few studies have explored the relationship between endometriosis and GH-PE. In a Medline search, we found only three studies with conflicting conclusions. Two studies reported that endometriosis was associated with increased [3] and decreased [21] risk of future GH-PE, while another study reported no association between the two pathologies [22]. Thus, the conflicting conclusions retrieved from these aforementioned studies with different research designs and samples are confusing. Besides, the major limitation in previous studies is that some of them were conducted employing a method of purposive sampling and small samples for cases and controls, which makes the conclusions questionable and less powerful. Moreover, some of the studies were completed using physician-identified inclusion criteria for endometriosis or GH-PE. Subjective recognition of the diagnosis made by physicians, rather than a stricter standard of diagnosis, may result in selection bias. These criteria could have a substantial confounding effect on the results of these studies. Furthermore, the conclusions of some studies are limited because the studies focused on patients with endometriosis who had undergone assisted reproduction techniques (ART) or in vitro fertilization (IVF) before they became pregnant. Therefore, it is important to elucidate the role of endometriosis as a predictor of subsequent GH-PE using a large nationwide population with stricter selection criteria to reach reliable conclusions on the relationship between endometriosis and GH-PE.

## Materials and methods

### Data source and sample

Started in 1995, the National Health Insurance (NHI) is a single-payer health insurance program. In Taiwan, the NHI covered 93% of the population in 1997, and the coverage increased to 99% at the end of 2010. In this study, we analyzed data from the NHI Research Database (NHIRD), a nationwide database that has been extracted from the claims data of the NHI program for research purposes. All of the data in the NHIRD are de-identified by scrambling the identification codes of both patients and healthcare providers. This database contains comprehensive information on inpatient and outpatient medical claims on anonymous insured patients, including demographic data, dates of clinical visits, disease diagnoses, and prescription records. In the NHIRD, International Classification of Diseases, 9th Revision, Clinical Modification (ICD-9-CM) codes are used for disease diagnosis, and NHI codes are used for procedures or treatments. The Longitudinal Cohort Dataset 2010 (Longitudinal Health Insurance Database, LHID2010), a data subset of the NHIRD is specifically for cohort studies; 1,000,000 individuals who were insured in 2010 were randomly sampled, and all of their claims data constitute the LHID2010. This study was an analysis of a nationwide population-based database, enrolling eligible women in the database of NHIRD rather than recruiting participants after obtaining their consents. Therefore there were no participants who had the willing to “participate” in or “withdraw” from the study. According to our experiences in using LHID for population-based studies [23], the main reasons for censored data were deaths and withdrawal of nationals from the NHI. This dataset has been confirmed to have no significant difference in age, sex, or healthcare costs compared with the whole population, which is composed of all beneficiaries in the NHI program.

### Ethics statement

According to the local regulations, the requirement for informed consents was waived as all claims data in the NHIRD were anonymized and de-identified before release. This study was granted a review waiver by the Institutional Review Board of Taipei Tzu-Chi Hospital, Taiwan (protocol number: 05-W02-044).

### Inclusion and exclusion criteria

We selected women previously diagnosed with endometriosis (ICD-9-CM codes 617.X) between 1998 and 2012 from the LIHD2010 dataset and designated these women as the exposed group after they were pregnant. Because the gold standard for diagnosing endometriosis is a surgical assessment by laparoscopy or laparotomy [2], for a valid diagnosis the condition of endometriosis should be tissue-proved and be confirmed by reviewing records of surgical treatment for endometriosis. Surgical treatment for endometriosis in this study was defined as surgery for adenomyosis (NHI codes 80402, 80415, 80420, 80425), adnexal endometrioma (NHI codes 80802, 80807, 80811, 80812), and excision of pelvic endometriosis (NHI codes 80014, 80029, 80031, 81014, 81032, 81033). Initial screening was performed to exclude women who had missing data in the medical record. The date of the first valid diagnosis of endometriosis was defined as index date.

The following exclusion criteria were used to analyze the risk of subsequent GH-PE (ICD-9-CM codes 642.X). We excluded women who were not diagnosed at a reproductive age. Because this study analyzed the relationship between endometriosis and subsequent pregnancy complications, we focused on reproductive-aged women during the study period. Consequently, women diagnosed at <15 or >45 years of age were excluded. Because we focused on GH-PE, the women who had chronic hypertension (ICD-9-CM codes 401.X-405.X) before 20 weeks gestation were not included or evaluated in the study. We also excluded women with GH-PE prior to the diagnosis of endometriosis. Furthermore, women with inconsistent diagnoses of endometriosis, which meant that there were discrepancies of ICD codes or medical records among the ambulatory medical records, operation notes, pathology reports, and admission records, were excluded during the survey. Because the NHI program covered 10 antenatal visits, almost all gravidas had regular antenatal visits. Therefore, if the woman had no record of prenatal visits 150 days before the diagnosis of GH-PE, the diagnosis was considered invalid. Moreover, if the diagnosis of GH-PE did not accompany reports of blood pressure or urine protein, the diagnosis was not considered valid. Based on a review of the medical records and laboratory test results, the diagnosis and coding of endometriosis and GH-PE are stricter and more reliable.

**Fig 1:**
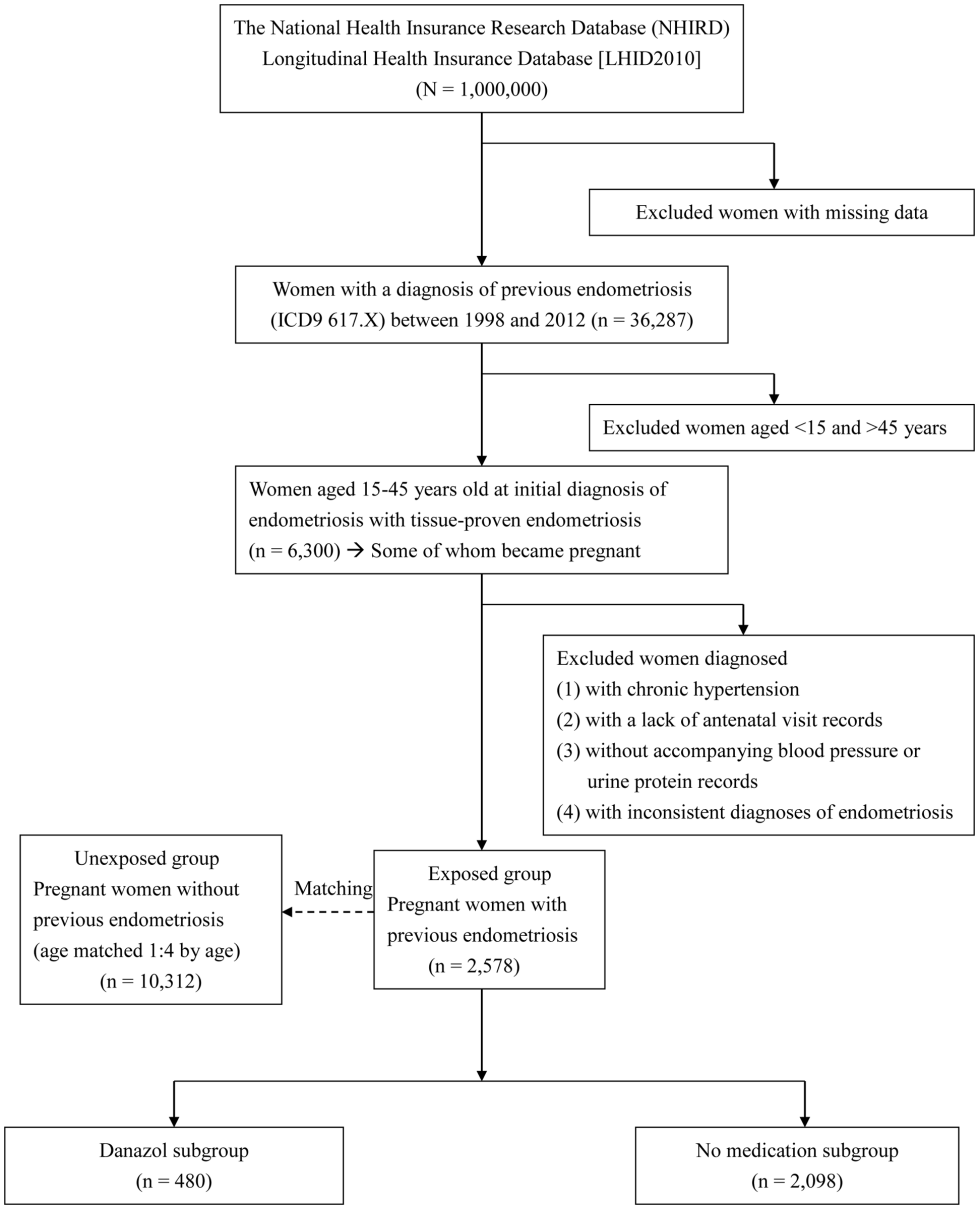
The flow chart of case inclusion and exclusion of women with previous endometriosis.

Since women who were <15 or >45 years of age at the time of diagnosis and who had inconsistent diagnoses were excluded to ensure the accuracy of the diagnoses, we selected beneficiaries without the diagnosis of endometriosis from the remaining women in the LHID2010 as the unexposed group. Each pregnant woman with a prior diagnosis of endometriosis (the exposed group) was age-matched to 4 pregnant women without previous endometriosis (the unexposed group). Every woman in the unexposed group were assigned an index date identical to that of the corresponding exposed group. We then identified every pregnancy in the exposed and unexposed groups and examined whether GH-PE had occurred during the pregnancy. As for women who had more than one pregnancy complicated with GH-PE, only the first occurrence of GH-PE was analyzed. Meanwhile, given that parity may influence on the risk of developing GH-PE, we also analyzed the first GH-PE at which pregnancy had occurred among the exposed and unexposed groups. Fig 1 shows the flow chart of case inclusion, exclusion, and classification.

In addition to investigating whether preceding endometriosis increased the risk of GH-PE, we further analyzed the effect of danazol, a common drug for treating endometriosis to determine whether danazol had any effect on reducing the occurrence of GH-PE. We then categorized patients with prior endometriosis into two subgroups according to danazol usage. In contrast to the patients without danazol use for endometriosis, the women who had taken danazol solely were defined as the danazol subgroup. In the current study, the danazol treatment was only included for analyses if it preceded the index pregnancy.

We analyzed the demographic characteristics such as age at the diagnosis of endometriosis, age at first pregnancy, parity (pregnancy with GH-PE), occupation, the degree of urbanization, economic status, and comorbidities, among women in the exposed and unexposed groups. We categorized occupation as white collar, blue collar, and retired/others. The degree of urbanization was classified into urban, suburban, and rural. Insurable wage was used as a proxy measurement of economic status. In the NHI system, the classification of economic status was represented by insurable wage (current exchange rate is US$ 0.0321 = New Taiwan Dollar [NTD] 1.00). Therefore, we divided economic status into 4 groups, as follows: insurable wage < NTD 20,000; NTD 20,000–40,000; ≧NTD 40,000; and other (spouse/dependents). Comorbidities as possible confounding factors included diabetes (ICD-9-CM codes 250.X), dyslipidemia (272.X), congestive heart failure (428.0X), ischemic heart disease (410.X-414.X), cerebrovascular disease (430.X-438.X), chromosomal anomalies (758.X and 317.X-319.X), and autoimmune disease. Autoimmune diseases included systemic lupus erythematosus (ICD-9-CM codes 710.0), systemic sclerosis (710.1), rheumatoid arthritis (714.0, 714.30–714.33), polymyositis (710.4), dermatomyositis (710.3), vasculitis (446.0, 446.2, 446.4, 446.5, 443.1, 446.7), Kawasaki disease (446.1), Behcet’s disease (136.1), pemphigus (694.4), Sjogren’s syndrome (710.2), Crohn’s disease (555.X), and ulcerative colitis (556.0–556.6, 556.8–556.9). In the current study, both previously identified risk factors (such as age at pregnancy, parity [pregnanies with GH-PE], diabetes mellitus, autoimmune disease) and confounding factors (such as occupation, urbanization, economic status, heart disease, cerobrovascular disease, and chromosome anoamlies) were considered and included for initial analyses. The factors which were statistically significantly different would be used in further regression models.

### Data analysis

SAS (version 9.4; SAS Institute, Inc., Cary, NC) was used for all data analyses. The chi-square test was used for categorical variables, and Student’s t-test was used for continuous variables. We analyzed the demographic characteristics including age at the time of initial diagnosis of endometriosis, age at first pregnancy, parity (pregnancy with GH-PE), occupational class (blue collar, white collar and retired and others), level of urbanization of residence (urban, suburban and rural), economic status, and comorbidities, among the exposed and unexposed groups. Logistic regression analysis was used to calculate odds ratios (ORs) and 95% conference interval (95% CI) for the risk of GH-PE with adjustment for age at the time of initial diagnosis of endometriosis, age at first pregnancy, occupation, urbanization, economic status and comorbidities. Additionally, Wald test was used to investigate the heterogeneity among the subgroups of danazol-treated analyses. A *p*-value < 0.05 was set as the level of significance.

## Results

Among the population of 1,000,000 residents who were insured in 2010, there were 492,423 males and 507,577 females. The latter included 250,804 females of reproductive age (age 15–45) and 256,773 females of non-reproductive age. Based on the inclusion and exclusion criteria, 6,300 women of reproductive age diagnosed with endometriosis between 1998 and 2012 were identified (Fig 1). Among these women, 2,578 (40.92%) had subsequent pregnancies and were eligible for further analysis. They were compared with 10,312 pregnant women without previous endometriosis. For every female in the exposed and unexposed groups, the follow-up duration for the occurrence of GH-PE was 15 years since the index date. In the current study, the mean and median follow-up duration was 7.24–7.35 years and 7.06–7.18 years, respectively. The main reasons for censored data were deaths of nationals (55/2,578 in exposed group and 158/10,312 in unexposed group) and withdrawal of nationals from the National Health Insurance (38/2,578 in exposed group and 227/10,312 in unexposed group). Table 1 compares the characteristics between the exposed and unexposed groups. The age in Table 1 provides the age (three age groups: 15–25, 26–35, and 36–45 years) of the women when endometriosis was initially diagnosed. During pregnancy, every woman with previous endometriosis (exposed group) was matched with 4 other women of the same age (unexposed group). For the exposed group, the average age at the time of initial diagnosis of endometriosis was 31.8 ± 5.8 years. Nearly 60% of these women were initially diagnosed with endometriosis between 26 and 35 years of age, followed by age 26.4% at 36–45 years, and age 14.2% at 15–25 years. The parities of pregnancies complicated with GH-PE were analyzed. For the exposed and unexposed groups, there were no differences in the ratios of primi-parity and multi-parity (p > 0.05). Compared with the unexposed group, a higher percentage (21.30%) of women in the exposed group had a high insurable wage (≥ NTD 40,000); a lower proportion (20.05%) of women had a low insurable wage (< NTD 20,000), and spouse/dependents had the lowest percentage of women (13.85%). The average age (30.1 ± 5.1 years) at first pregnancy in the exposed group was older than that (29.3 ± 4.8 years) in the unexposed group (*p* < 0.0001). Compared with the unexposed group, women in the exposed group were more likely to be white-collar workers (*p* = 0.0006), to reside in urban areas (*p* = 0.0298), and to have a higher level of economic status (*p* = 0.0008). In addition, they were more likely to have dyslipidemia (*p* = 0.0155) and autoimmune disease (*p* = 0.0153) (Table 1).

**Table 1:** Characteristics of women with and without previous endometriosis.

	Exposed Group: Women with endometriosis (n = 2578)		Unexposed Group: Women without endometriosis (n = 10312)		Statistics		
	N	%	N	%	OR	95% CI	P-value
**Age of diagnosed**	31.77±5.76		31.77±5.76				1.0000
15–25	366	14.2	1464	14.2			
26–35	1532	59.43	6128	59.43			
36–45	680	26.38	2720	26.38			
**Age at first pregnancy**	30.08±5.07		29.30±4.79				<0.0001***
**Pregnancies with GH-PE (parity)**							0.7078
1st	88	88	149	88.69			
2nd	12	12	18	10.71			
3rd or more	0	0	1	0.60			
**Occupation**							0.0006**
White collar	1444	56.01	5514	53.47			
Blue collar	596	23.12	2274	22.05			
Retired and others	538	20.87	2524	24.48			
**Urbanization**							0.0298*
Urban	1737	67.38	6689	64.87			
Suburban	709	27.5	2994	29.03			
Rural	132	5.12	629	6.10			
**Economic status**							0.0008*
Insurable wage < 20,000 NTD	517	20.05	2255	21.87			
Insurable wage 20,001–40,000 NTD	1155	44.80	4515	43.78			
Insurable wage ≧ 40,000 NTD	549	21.30	1915	18.57			
Other (Spouse/dependents)	357	13.85	1627	15.78			
**Comorbidities**							
Diabetes mellitus	79	3.06	263	2.55	1.21	0.94–1.56	0.1464
Dyslipidemia	139	5.39	442	4.29	1.27	1.05–1.55	0.0155*
Congestive heart failure	6	0.23	10	0.10	2.40	0.87–6.62	0.0799
Ischemic heart disease	62	2.40	205	1.99	1.21	0.91–1.62	0.1836
Cerebrovascular disease	47	1.82	190	1.84	0.99	0.72–1.36	0.9477
Autoimmune disease	204	7.91	677	6.57	1.22	1.04–1.44	0.0153*
Chromosomal anomalies	5	0.19	23	0.22	0.87	0.33–2.29	0.7766

*p < 0.05, **p < 0.001,***p < 0.0001, by chi-square test or student t test, as appropriate.

Data are expressed as the number (%) or mean ± standard deviation, as appropriate.

Table 2 shows the results of the risk analysis of the association between endometriosis and subsequent GH-PE. During the study period, 100 cases of GH-PE were noted among 2,578 patients with preceding endometriosis while 168 cases were observed among 10,312 patients without preceding endometriosis. We demonstrated that GH-PE occurred more frequently among women in the exposed group compared with women in the unexposed group (3.88% vs. 1.63%, *p* < 0.0001). After adjustment with covariates, logistic regression analysis revealed that a history of endometriosis is an independent and significant risk factor for GH-PE (adjusted OR = 2.27; 95% CI: 1.76–2.93).

**Table 2:** Risk analysis between endometriosis and subsequent GH-PE.

	No GH-PE		GH-PE		Statistics	
	N	%	N	%	Crude OR (95% CI)	Adjust OR (95% CI)
**GROUP**						
Unexposed group (n = 10,312) Women without endometriosis	10144	98.37	168	1.63	Reference	Reference
Exposed group (n = 2,578) Women with endometriosis	2478	96.12	100	3.88	2.48** (1.90–3.13)	2.27** (1.76–2.93)
Subgroup in exposed group						
No medication group (n = 2,098)	2013	95.95	85	4.05	2.55** (1.96–3.32)	2.48** (1.89–3.25)
Danazol group (n = 480)	465	96.88	15	3.13	1.95* (1.14–3.33)	1.49* (0.86–2.56)

*p < 0.05, **p < 0.0001, Odds ratio (OR) and 95% confidence intervals (95% CI) are calculated by logistic regression analysis, as compared to the reference group.

Adjusted for age at the time of initial diagnosis of endometriosis, age at first pregnancy, occupation, urbanization, economic status, dyslipidemia and autoimmune disease.

We further determined whether danazol use for women with previous endometriosis reduced the risk of developing GH-PE (Table 2). Moreover, we performed Wald test to investigate the heterogeneity among these subgroups. It shows no significant difference among them (*p* = 0.1151). Among the 2,578 women with endometriosis, 480 (18.62%) had taken danazol solely for endometriosis. For danazol-treated and non-danazol-treated subgroups, the incidences of GH-PE were 3.13% (15/480) and 4.05% (85/2,098), respectively. Although the risk for subsequent GH-PE was lower (adjusted OR = 1.49; 95% CI: 0.86–2.56) after receiving danazol treatment than average (adjusted OR = 2.27; 95% CI: 1.76–2.93) for women with preceding endometriosis, the reduction of risk was not statistically remarkable for danazol-treated (adjusted OR = 1.49) vs. non-danazol-treated (adjusted OR = 2.48) subgroups (p heterogeneity = 0.12).

## Discussion

In the current study, women with prior endometriosis have a higher incidence of GH-PE in subsequent pregnancies compared to women without endometriosis (3.88% vs. 1.63%, *p* < 0.0001). Further analysis shows that preexisting endometriosis is an independent and significant risk factor (adjusted OR = 2.27; 95% CI: 1.76–2.93) for GH-PE. Women with preceding endometriosis are associated with a higher risk of developing GH-PE in subsequent pregnancies and require closer surveillance for maternal and fetal well-being. After confirmation of preexisting endometriosis, appropriate information and suggestions should be provided to pregnant women at-risk to facilitate earlier intervention or specialty referral. When affected women become pregnant, monitoring and preparation should be intensified before and during delivery to avoid obstetric complications due to the increased risk of GH-PE. In addition, the risk of subsequent GH-PE was not significantly reduced for women with endometriosis who received danazol treatment.

In 2007, Brosens et al. reported that endometriosis was associated with a decreased risk of PE [21]. Their conclusion is inconsistent with our findings (Table 2), which demonstrate an increased risk of GH-PE among gravidas with preceding endometriosis. However, several methodological attributes of their study differed from ours, including a smaller sample size, low response rate for questionnaires, and possible recall and selection bias, which could have accounted for the differences between the results [3]. Nonetheless, our findings are similar to those of another large-scale study [3].

The factors influencing the association between endometriosis and GH-PE are multiple and complicated. During pregnancy, maternal uterine spiral arteries (SAs) are remodeled from minimal-flow, high-resistance vessels into larger diameter vessels with low resistance and high flow. This process is completed via the combined action of fetal extravillous trophoblasts (EVT) and decidual NK cells, which accumulate around SAs before trophoblast invasion [24]. GH-PE originates from a combined condition of poor placentation and placental ischemia and insufficiency [12, 19, 25]. In addition, activated macrophages within the uterus and placenta and peripheral helper T cells produce cytokines, including TNF-α, IL-6, and IL-7, which may predispose to PE [25–27]. Investigators have reported macrophage-mediated apoptosis of EVT has been associated with GH-PE [28]. We postulate that the elevated risk of GH-PE may result from activation of macrophages and modulation of NK cells, which are estrogen-mediated immunologic responses in women with endometriosis [2]. This assumption is supported by similar findings in both endometriosis and GH-PE, including activation of macrophages, increased production of cytokines such as IL-6, TNF-α, lipid peroxidation, and growth factors, and modulated NK cell activity [2, 25, 29, 30]. Furthermore, researchers have reported that alterations in the inner myometrium occurring in women with endometriosis and adenomyosis may be at the root of defective remodeling of the myometrial SAs from the onset of decidualization, which results in vascular resistance, increased risk of defective deep placentation, and subsequent GH-PE [31]. Another possible explanation for our findings is that in women with preexisting endometriosis, the epithelium and stromal cells are under a status of chronic inflammation and oxidative stress [2, 3, 30], which can induce GH-PE [26, 29, 30, 32] and can be found in affected patients.

Statistically, the use of danazol for endometriosis did not reduce the risk of future GH-PE. Since the growth of ectopic endometrial cells and tissues is estrogen-dependent [1, 2], the use of danazol has multiple effects on the endometrium and endocrine systems. Danazol has an androgenic effect, decreases ovarian estrogen production, and exerts a direct inhibitory effect on endometrial growth [33–35]. Low-estrogen status of endometrium can also be induced using other medications included progesterone, which suppresses the action of estrogen receptor, and GnRH analogues, which inhibit the function of hypothalamic–pituitary–gonadal axis. Moreover, via a mechanism of lymphocyte-mediated cytotoxicity, danazol modulates the immunologic function of stromal cells [34–36]. One study has confirmed that danazol inhibits aromatase activity of endometriosis-derived stromal cells by a competitive mechanism [35]. Thus, the estrogenic effect on endometrial cells may be reversed by danazol, which further prevents stromal invasion and angiogenesis of tissues that can be found in both patients with endometriosis and GH-PE. Nevertheless, the reduction of risk for subsequent GH-PE did not reach a statistical significance for the danazol-treated subgroup. A possible interpretation of this result of the subgroup analysis is that danazol was often used for patients with endometriosis of higher severity rather than those with no or minor symptoms. Thus, the benefit of protecting women from GH-PE by the use of danazol may be offset by the aggravating effect of endometriosis of more severity that leads to the use of danazol. Detailed discussions of the possible mechanism is beyond the scope of the current study, but we believe that further efforts should be made to explore the etiology that underlies GH-PE in women with preexisting endometriosis and also relevant medications.

Our study has several strengths including a large sample size, sound sampling method, and stricter selection criteria for women diagnosed with endometriosis or GH-PE. One advantage of our study is that the sample was retrieved from the database of a general survey of a national population rather than from purposive sampling or women who have undergone ART or IVF. The sample size of the current study is large among all studies of its kind to investigate the prevalence of GH-PE for women with preceding endometriosis. Therefore, our results are robust due to minimization of possible errors that originate from the sampling process. Moreover, all diagnoses for women with endometriosis or GH-PE were made by objective surgical or pathologic confirmation, physical examination, and laboratory tests rather than by the subjective judgment of a physician, compared with those made in previous studies [3, 21, 22]. All biases resulting from the samples, the selection, the investigator, and the measuring process were minimized by the methods and criteria used. Additionally, we analyzed the effect of danazol treatment on subsequent GH-PE, which has not been previously reported [3, 21, 22].

This study has several inherent limitations despite its strengths. First, it was not easy to precisely define a medical condition from administrative data. Using ICD codes alone did not entirely reflect the real status of clinical conditions. Therefore, we not only used ICD codes to identify cases, but also considered reports of physical examination, laboratory testing, and surgeries as essential inclusion criteria to raise the appropriateness of the case definition. Under the NHI system, administrative data were collected for the purpose of reimbursement. Thus, the existing data could have been inconsistently collected over time. This inconsistency could have affected the results to some extent. Some demographic characteristics and data of medication were lacking in NHIRD, such as body mass index (BMI), smoking habit, social status, marital status, and self-paid drugs and instruments such as oral contraceptives, depo provera, intra-uterine devices, Lupron, or other GnRH-agonists, which were not covered under the NHI system in Taiwan. Thus, we were unable to investigate the contributing influence of these factors or non-danazol treatments. Because height and body weight of every covered resident, self-paid drugs and instruments were not routinely recorded in this national health database of Taiwan, to obtain such data was not possible. Moreover, although the power of the analysis of subgrouping is adequate (more than 450 women in each subgroup), we could not obtain the information on danazol dosage and duration, which was a limitation of the national database and could have affected the study result. Due to the ethnic homogeneity of Taiwan, the results do not reflect the effect of the ethnicity on the association between endometriosis and GH-PE. Finally, the results might not be generalized to other ethnic populations. In this longitudinal study, factors that varied with time and changes in medical policies could not be controlled.

In conclusion, this nationwide, population-based study demonstrated that pregnant women with preceding endometriosis have a more than two-fold increased probability of developing GH-PE compared with women without endometriosis. Statistically, the use of danazol for endometriosis does not reduce the risk of future GH-PE. We recommend that obstetricians and gynecologists should be aware of the increased risk of future GH-PE in women with previous endometriosis. When at-risk women become pregnant, monitoring and preparation should be intensified before and during delivery to avoid obstetric complications due to the high risk of GH-PE. Although this study has provided helpful information, many unknown aspects regarding GH-PE warrant further investigation.
